# A comparison of health communication effectiveness and the improvement of management strategies: taking two Chinese traditional medicine hospitals’ WeChat public accounts as examples

**DOI:** 10.1186/s12913-020-05901-3

**Published:** 2020-11-20

**Authors:** Jie Wang, Lanting Wu

**Affiliations:** 1 Faculty of communication, Hangzhou Dianzi University, Hangzhou, Zhejiang 310008 People’s Republic of China; 2 Library, Anhui Agricultural University, Hefei, Anhui 230000 People’s Republic of China

**Keywords:** Communication effect, Health communication, Social media, WeChat public account

## Abstract

**Background:**

In the light of “Internet plus”, hospitals are following the trend of using mobile internet and adopting a strategy of spreading public health knowledge through mobile terminals. WeChat is a social media with the largest number of users in China. Its public account has become the most popular service among the public.

**Methods:**

We examine the health communication of medical institutions on social media platforms. The WeChat public accounts of Zhejiang Provincial Chinese Traditional Medicine Hospital and Jiangxi Provincial Chinese Traditional Medicine Hospital were taken as cases to measure the communication effect from the following dimensions: update interval, content positioning and design, numbers of clicks and likes as well as topic types.

**Results:**

The update interval of WeChat public account of Jiangxi Provincial Hospital of Traditional Chinese Medicine was regular, compared with that of the other hospital. The accounts of the two hospitals both set up special sections to facilitate patients to obtain medical services online. There is an extremely significant difference between the two hospitals’ mean numbers of clicks (*p* < 0. 001), compared with no significant difference between their mean numbers of likes. One-way analysis of variance suggests the type of topic on posts is significantly correlated with the number of clicks. Moreover, there is an extremely significant difference between public health knowledge and news propaganda.

**Conclusion:**

The development of hospitals’ WeChat public accounts can promote people’s health and equity in accessing medical information and service, and also boost “Internet plus health care” service. The topic type of hospital’s news publicity is paid a relatively lower attention by users. Therefore, hospitals’ WeChat public accounts need to adjust their strategy from propaganda-oriented to users-centered, with topic planning and posts designed to fulfill users’ needs.

## Background

In the context of “Internet plus”, one of China’s national strategies is to encourage industrial innovation as well as promoting cross-border integration, aiming at using information and communication technology to make the internet deeply integrated with various traditional industries. It will improve people’s wellbeing and push forward social development and innovation [1]. In the medical industry, “Internet plus” means “Internet plus health care” service. The trend of disseminating public health information is becoming widespread through mobile terminals.

As a social media, WeChat has the largest number of users in China. It has global competitors including Facebook and WhatsApp. Gai thought WeChat has become the ultimate combination of Facebook, Twitter, WhatsApp and Zynga which provides payment, reservations, booking, games, instant messaging (IM) and other services [2]. Similarly, Yang et al. argued WeChat has developed into an all-encompassing behemoth of IM, payment, micro-commerce, applet, public account [3]. In 2017, the number of WeChat public accounts reached 10 million with 3.5 million active public accounts, and the number of active users stood at 719 million people [4]. In 2019, the number of WeChat monthly active users exceeded 1.15 billion people [5].

WeChat public account is the most popular function through WeChat platform. Health information is one of the hottest topics with the most views, pushes and reposts [6]. In 2017, the cumulative clicks of health WeChat public accounts in Shanghai was more than 54.81 million, making them the main channel for citizens to conveniently obtain information of health matters and hospital services [7]. During the outbreak of COVID-19, many hospitals went to great lengths to provide medical service online to reduce crowd gathering and cross infection. For example, Xiamen Children’s Hospital in Fujian Province, developed online consultation service on the WeChat public account [8], while Cardiovascular Hospital of Xiamen University in Fujian Province developed the medical records mailing service on the WeChat public account [9].

WeChat public account includes enterprise account, service account and subscription account. The service account and the subscription account are open to the public and mainly for customer service and information release, while the enterprise account is for communication among employees and enterprises’ management, which has been integrated into some enterprise apps currently [10]. There are some differences between a subscription account and a service account. The former is usually used by individual users and organizations, while the latter is more welcomed by organizations. They are also different in the display interface of mobile terminals. A subscription account is folded on the list page, while the other one appears directly on the chat page. Meanwhile, the former can be upgraded to a service account for more advanced features such as geo-localization. WeChat public account provides an interactive platform between organizations and users [11].

Mass media and professional medical institutions are the main bodies that disseminate health information in China [6]. Public hospitals, especially first-class hospitals, with the most adequate medical staffs, resources, and technology, are the main providers of health information. It is obvious that there is asymmetry of health information between social crowds and hospitals. Social media can be used as a health promotion strategy [12]. Many of the hospitals in China have registered WeChat public accounts. For example, almost all the first-class hospitals in Beijing, Shanghai and Guangzhou had created WeChat Public accounts by 2016, which possess the most high-quality medical resources in China [13]. Public health institutions should pay attention to and make good use of social media to achieve the goal of health communication [14].

The feedback of the receiver to the sender is an essential part of communication process. The communication effect, as a strong reference to guiding content production, means to what extent communication activities can realize the intention of the sender. Readability is important on communication. Readability refers to the quality of being easy and enjoyable to read, usually measured by Flesch Reading Ease, Flesch-Kincaid Grade Level, and Gunning Fog Index. These formulas measure texts’ readability, including sentence length and vocabulary familiarity based on semantic difficulty and syntactic complexity [15]. Both reducing the difficulty of understanding and improving humanity of the information can raise readability [16]. According to the use and gratification theory, users actively select the types and content to satisfy their needs [17]. The medium that provides the most satisfying content will be used more often than others. Thus, it is necessary to evaluate and understand the audience’s cognition, attitude on types and contents of media.

However, research in online health communication mostly focuses on the traditional online community and microblogging platforms, while the current empirical analysis of WeChat with public communication functions is very scarce [6]. Although WeChat is one of the most important social media platforms in China, there is almost no empirical research in it [18]. A few studies focus on the dissemination of health information on WeChat and believe that the communication of health information is influenced by social characteristics of information content. The social traits of practical values and the emotional traits of information affect the spread of health information [19]. Compared with the research about popular social media such as Facebook in western countries, there is a lack of research on the social impact of WeChat in the international community [11].

Health knowledge such as daily health care, disease prevention and treatment need to be released by professional medical organizations to ensure the credibility of information. Hospitals’ WeChat public accounts can help spread health information, boost public health awareness and build the public welfare image for the hospitals. The research questions in this paper are as follows: what the positioning and functions of hospital WeChat public accounts are? How to improve their communication effect? This research evaluates accounts’ communication effect between hospitals and users and explores the ways these accounts can best promote public health.

## Methods

We selected WeChat public accounts of two Chinese Traditional Medicine hospitals as cases-Zhejiang Provincial Chinese Traditional Medicine Hospital and Jiangxi Provincial Chinese Traditional Medicine Hospital. The WeChat public account of Zhejiang Provincial Chinese Traditional Medicine Hospital is a service account and that of Jiangxi Provincial Chinese Traditional Medicine Hospital is a subscription one. The hospitals are located respectively in the eastern and middle regions of China. Both are first-class hospitals. In China, First-class hospitals refer to the hospitals at ranking of 3A, with class three and grade A, according to the “Rules to be in charge of hospitals by grade”, formulated by the Ministry of Health of China in 1989. These comprehensive hospitals are government-funded and equipped with more than 500 beds, being responsible for medical research and teaching tasks.

Jiangxi Provincial Chinese Traditional Medicine Hospital’s public account is one of the most popular ones among hospitals in China. It has been reported by Health News [20]. Health News is the most influential national health newspaper under the Ministry of Health of China. We selected posts from WeChat public accounts of two hospitals between October 19, 2018 and November 30, 2018. We took samples of posts released by hospitals’ public accounts in winter time. It is because taking winter cream formulae is very popular among Chinese. Practitioners of Traditional Chinese Medicine believe that winter is the best season to take cream formulae which can strengthen people’s health. Thus, the time of sample selection was typical.

In this research, the two hospitals’ accounts were taken as cases to measure the communication effect. IBM SPSS Statistics 25.0 software was used for the data analyses. Descriptive analysis was used to compare the update interval. T-tests were used to compare the means of the numbers of clicks, as well as the means of the number of likes. Analysis for topic types coding were grouped into six categories according to the content of the posts. One-way analysis of variance (ANOVA) was adopted to analyze the correlation between the number of clicks and the type of topics, as well as the correlation between the numbers of likes and the type of topics.

## Results

### Update interval of posts

During this period, the WeChat public account of Jiangxi Provincial Hospital of Traditional Chinese Medicine updated 37 times and released 68 posts. However, the content of the posts on November 16 was deleted by the publisher, and the type of posts released on November 9 was public health knowledge. This was assessed as false information by the third party. The actual number of posts was 66 when two posts with missing data were excluded. Zhejiang Provincial Hospital of Traditional Chinese Medicine updated 7 times with a total of 17 posts pushed. The update time was irregular, and the update interval ranged from one to 15 days. The update interval of the WeChat public account of Jiangxi Provincial Hospital of Traditional Chinese Medicine was relatively regular, usually from one to 3 days, mostly updated on daily basis (Table 1).

**Table 1:** Descriptive statistics

WeChat public account	Number of posts	Updates	Update interval (days)	Mean interval
Jiangxi Provincial Hospital of Traditional Chinese Medicine	66	37	1–3	1.1
Zhejiang Provincial Hospital of Traditional Chinese Medicine	17	7	1–15	6

### Content positioning and design

Posts of WeChat public account consisted a main post and a secondary post. The main post is at the top of the post page, in form of catchy videos or pictures, which easily attracts attention. The secondary post lies on the relative edge position and uses a text title as the main body, and an image as an auxiliary. The comment section is set at the bottom of the post page. If the followers make some comments in this section, the editor can reply to them in a very short time.

The design of the comment area is aimed at promoting interactions between hospitals and the public, and between members of the users. However, sometimes, titles of the posts on the homepage of Zhejiang Provincial Hospital of Traditional Chinese Medicine is too long that they cannot be showed in a full view. Thus, it is not convenient for users to easily browse the contents. On WeChat public accounts, the higher readability a post has, the better the communication effect can be obtained and vice versa.

The columns at the bottom of the page in Zhejiang Provincial Hospital of Traditional Chinese Medicine’s WeChat public account are divided into three sections including “medical navigation”, “healthy steward” and “my information”. Jiangxi Provincial Hospital’s account is divided into “surfing online”, “medical guide” and “self-service” sections. The two hospitals’ accounts both have special sections for patients to search doctors, provide online inquiry information, make appointments for doctors and registrations, mobile payment and obtain medical reports online.

### Clicks and likes

WeChat public account provides a dashboard to display the number of clicks and likes of each post. The total number of clicks of Zhejiang Provincial Hospital of Traditional Chinese Medicine’ account is 55,640, and the total number of likes stands at 552. Among them, the average click is 3272.9, and the average likes is 32.5. The pike of clicks is 11,776, and the least click is 615. By contrast, the total number of clicks for the WeChat public account of Jiangxi Provincial Hospital of Traditional Chinese Medicine is 739,839, and the total number of likes is 2301. Its average click is 11,209.7, with an average value of 34.9, a maximum number of clicks of all the samples up to 42,716, and a minimum number of clicks 843 (Table 2 and Table 3).

**Table 2:** Numbers of clicks

Clicks of WeChat public account	N	Min	Max	^−^X	SD
Jiangxi Provincial Hospital of Traditional Chinese Medicine	66	843	42,716	11,209.7	7951.1
Zhejiang Provincial Hospital of Traditional Chinese Medicine	17	615	11,776	3272.9	2643.9

**Table 3:** Numbers of likes

Likes of WeChat public account	N	Min	Max	^−^X	SD
Jiangxi Provincial Hospital of Traditional Chinese Medicine	66	4	110	34.9	23.2
Zhejiang Provincial Hospital of Traditional Chinese Medicine	17	5	114	32.5	25.5

According to data analysis, the total number of clicks and likes of the public account of WeChat in Jiangxi Provincial Hospital of Traditional Chinese Medicine are both higher than those of Zhejiang Provincial Hospital of Traditional Chinese Medicine. Furthermore, the average numbers of clicks and likes of the former are also higher than the latter.

According to the independent samples test results of the mean number of clicks, there is a significant difference between the two hospitals (*p* < 0.001). The number of clicks shows to what exact extent that users are attracted by the health information. In terms of clicks, the communication effect of the WeChat public account of Jiangxi Provincial Hospital of Traditional Chinese Medicine is significantly higher than that of the Zhejiang Provincial Hospital of Traditional Chinese Medicine. As for the t-test results of the mean number of likes, due to the overall variance homogeneously, sig = 0.711, there is no significant difference between the two hospitals.

### Topic type

We divided the content of the posts into six categories: public health knowledge, winter cream formulae, activity notice, doctor information, talent recruitment and news propaganda. In order to maintain the reliability of coding, we invited a postgraduate student with a management background to code all the data. The coding consistency is 0.98, which meets the minimum reliability requirement of 0.85, accepted generally [21]. The themes of the WeChat public account of Zhejiang Provincial Hospital of Traditional Chinese Medicine include the above six categories, while the themes of the WeChat public account of Jiangxi Provincial Hospital of Traditional Chinese Medicine cover three categories: public health knowledge, news propaganda and winter cream formulae. Therefore, the WeChat public accounts of the two hospitals are in line with the timing of seasonal changes. The introduction and usage of the winter cream formulae have been included in the posts, which shows that the WeChat public account has two functions: One is for Traditional Chinese Medicine knowledge dissemination and the other is for sales promotion of winter cream formulae.

**Fig. 1:**
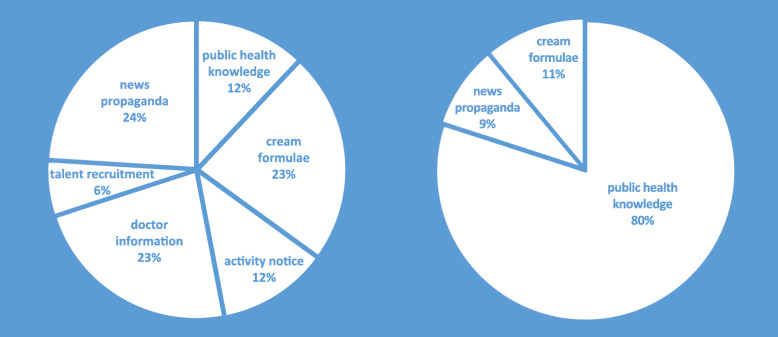
Topics distribution on WeChat public account of the two hospitals

According to the statistics, public health knowledge accounted for 12%, while news propaganda accounted for 24% of the posts released by Zhejiang Provincial Hospital of Traditional Chinese Medicine. The public health knowledge accounted for 80% of all the posts by Jiangxi Provincial Hospital of Traditional Chinese Medicine, while the hospital news category accounted for 9%. The topic distribution of the posts of the two hospitals is shown in Fig. 1. The left diagram is about WeChat public account of Zhejiang Provincial Chinese Traditional Medicine Hospital and the right one is about that of Jiangxi Provincial Chinese Traditional Medicine Hospital.

To explore the relations between the number of clicks and the type of topics, One-way ANOVA is performed and the results show there is a significant difference between different topic categories of posts Groups (*p* < 0.001). It suggests the topic type of posts is significantly correlated with the number of clicks. Based on the outcome, we take clicks as the dependent variable and then conduct multiple comparisons (Table 4).

**Table 4:** Clicks and likes of the topics

Topic	N	Mean of clicks	Mean of likes
Public health knowledge	55	12,379	34
Cream formulae	11	5066	37
News propaganda	10	3662	43
Doctor information	4	1404	4
Activity notice	2	2432	2
Recruitment	1	11,776	38

As the talent recruitment category is an isolated case in samples, it is not performed among the multiple comparison. According to the above table, the post topic is related to the number of clicks. There appears a significant difference in the number of clicks as topics vary from public health knowledge, to the cream formulae and to the doctor’s information, especially between topics of public health knowledge and news propaganda. By contrast, there is no significant difference in the number of clicks between public health knowledge topics and activity notice. The theme of activity notice includes diagnosis and recruitment of patients. Activity notice means an instant communication between doctors and patients. The results indicate that users have a high interest in health information which contains public health knowledge and activity notice (Table 5).

**Table 5:** Multiple Comparisons of clicks for topics

Topic type	Topic type	Significant
Public health knowledge	cream formulae	**
	news propaganda	***
	activity notice	0.101
	doctor information	**

* *p* < 0.05, ** *p* < 0.01, *** *p* < 0.001

To explore the relationship between numbers of likes and different topics, we take numbers of likes as the dependent variable. One-way ANOVA was conducted for different topic categories of posts. It indicates that there is no significant difference in numbers of likes for different post topics.

## Discussion

Readability will affect the users’ acceptance of the content and it is also a core indicator of assessing the usefulness of information [22]. In the visual design of posts, important content is in the center of the homepage in form of pictures or videos. In terms of symbol selection, public accounts of the two hospitals both use multi-mode symbols including video, audio, micro-video, text, pictures and animation, which can improve the readability of information. The layout of the front pages follows the principle of simplicity and clarity, and is easily accessed.

In terms of the content, content orchestration increases the readability. The primary push and the secondary push work together to promote clicks. Hyperlinks in different posts help to increase clicks and the exposure of information. The WeChat public account of Jiangxi Provincial Hospital of Traditional Chinese Medicine has set hyperlinks of related topics at the end of each post, allowing users to move to other posts more easily.

Moreover, the post content keeps in line with the seasonality of disease prevention. If it can provide specific treatment information for the specific disease, it will be more useful. The content that combines hot events and topics can further popularize health information. For example, the post with the highest click 42,716 was released by Jiangxi Provincial Chinese Traditional Medicine Hospital, named “Li Yong died of cancer. May the deceased rest in peace and the living be healthy”. Li Yong was a famous host working for China’s state television station whose death sparked big social response. The post aims at educating the people on cancer prevention knowledge through this case. By taking a celebrity’s case as an example, the post can popularize disease prevention knowledge and raise public awareness of keeping physical and mental health with better effect.

Besides, it is necessary to establish error correction mechanism to ensure the accuracy and reliability of health information. False information will undermine the public’s trust for hospitals and lead public’s wrong assessment of health information [23]. Correct health information helps to realize medical organizations’ social responsibility and establish social credibility of hospitals’ WeChat public accounts. For example, a post concerning health knowledge by Jiangxi Provincial Hospital of Traditional Chinese Medicine was evaluated as untrue by a third-party, named *Clove doctor*, then the post was deleted immediately. Preventative health care knowledge belongs to professional medical knowledge. Peer review and mutual supervision are conducive to maintaining the accuracy and authority of health information on social media.

The public accounts of the two hospitals popularize public health knowledge and provide various online medical services. In China, patients can go to a big hospital directly for medical treatment and the hospital does not require all the patients to make an appointment in advance. This brings some side effects. Due to large numbers of patients, it takes a long time to register. Meanwhile, the outpatient service system may find it challenging to arrange patients to register on the spot [24]. The two hospitals in our research take WeChat public accounts as a tool to solve this problem. To make an appointment online, users are required to fill in personal information including ID card numbers and mobile phone numbers in the hospital’s WeChat public account, through which the hospitals can obtain contact details of users. In fact, Appointment and registration service through hospitals’ WeChat public accounts is the most popular function embraced by users [13].

In conclusion, as public hospitals, the two hospitals do not focus on the sales promotion or see WeChat public accounts as marketing platform. Health information can boost chances for patients to participate in medical decision-making process, which can enhance the mutual trust between doctors and patients [25]. If users trust public accounts, they will accept their information more positively [26]. Keeping away from commercials will increase public accounts’ credibility among users.

The difference between the mean number of clicks of the two hospitals is partly ascribed to the stable and shorter update interval of posts from one hospital, which leads to a greater number of posts and maintains the readers group. Following hospitals’ public accounts shows that users are interested in them. The number of clicks is positively related to the number of users and posts. If the user number is constant, then the more posts, the more clicks. Posts updated regularly will help cultivate users’ habits of clicking. The public account of Jiangxi Provincial Hospital of Traditional Chinese Medicine is updated with a short interval, which contributes to establishing and cultivating stable connections with users, resulting in its improving communication effect.

Difference in the numbers of clicks and no significant difference in the number of likes of the two hospitals’ accounts indicate users’ click behavior of online health information can’t build a close connection between them and hospitals. It also displays a lack of offline interaction based on actual interpersonal relationships between providers and users. It can be explained as follows:

Generally, different from clicks, likes are one of users’ evaluations of health information, positive feedbacks and emotional response to the information providers, which shows users’ more participation involved. First, some researchers analyzed consumer behavior on mobile short video social platforms and found that users’ behavior of click can only meet their needs for functions of information, but does not trigger their emotional response. Besides, a lack of interaction can easily lead to user churn. Interactive behavior is conducive to improving users’ satisfactions [27]. Second, health information based on real interpersonal relationships is easier to be accepted and forwarded [28]. The research on health communication on Weibo shows topic types don’t largely affect users’ comments and likes. Comments and likes are sometimes irrelevant to the arguments. The user’s likes on social media are a behavior that is biased towards the real connections among private individuals and does not pay attention to the full presentation of arguments [29]. To improve the effectiveness of communication, it is necessary for public accounts to combine online information and offline interactive activities.

Interactivity is one of the main characteristics of new media. Activities offline should be combined with the posts in the hospital’s WeChat public account, which can promote emotional connections between organizations and users. This will increase users’ loyalty to the account. For example, in late October 2018, the public account of Jiangxi Provincial Hospital of Traditional Chinese Medicine launched a post about cream formulae. The activity in the post displayed an interactive campaign. It is said the top 10 users who give positive comments each day in the chronological order have priority to see and choose a doctor. Interactions between hospitals and users can encourage users to actively participate and share in a virtual community, as well as achieving the goal of better community engagement. Online communication and offline activities will have a synergistic effect on improving the popularity of hospital WeChat public accounts.

According to the analysis of the information contents and topic types, hospital’s news publicity of technical strength, the alleviation of poverty, or the administrative official activities, are not popular among users. For example, in posts by Jiangxi Provincial Hospital of Traditional Chinese Medicine, the one that got the lowest number of clicks is about hospital management’s alleviation of poverty in rural areas. This only meets the hospital’s needs of propaganda, while health knowledge meets the public’s real demand for health information.

### Limitations

The research is based on data of the two public hospitals. Besides, data collection time was limited.

## Conclusion

Hospital’s news publicity is paid a relatively lower attention by users. Therefore, the operation of hospitals’ WeChat public account needs to adjust the propaganda-oriented content strategy and truly focus on the users’ needs.

From the perspective of users, they long for health information. Obtaining information from the WeChat public accounts is free. The existing high-quality resources of medical information and services are mostly concentrated in urban areas in China. For people in rural areas, it’s much more difficult to access them. WeChat hospital public accounts can break geographical restrictions and provides health resources for people in need. In summary, the development of WeChat hospital public accounts can promote people’s health and the equity in accessing medical information and service.

## Data Availability

The datasets during and/or analyzed in the current study are available for the corresponding author on reasonable request.

## Abbreviations

ANOVA: Analysis of Variance
IM: Instant Messaging
